# A qualitative examination of barriers and facilitators of pediatric enhanced recovery protocol implementation among 18 pediatric surgery services

**DOI:** 10.1186/s43058-022-00329-8

**Published:** 2022-08-18

**Authors:** Teaniese L. Davis, Willemijn L. A. Schäfer, Sarah C. Blake, Sharron Close, Salva N. Balbale, Joseph E. Perry, Raul Perez Zarate, Martha Ingram, Jennifer Strople, Julie K. Johnson, Jane L. Holl, Mehul V. Raval

**Affiliations:** 1 Center for Research and Evaluation, Kaiser Permanente Georgia, Atlanta, GA USA; 2 Center for Health Services and Outcomes Research, Institute of Public Health and Medicine, Northwestern University Feinberg School of Medicine, Chicago, IL USA; 3 Surgical Outcomes and Quality Improvement Center, Department of Surgery, Northwestern University Feinberg School of Medicine, Chicago, IL USA; 4 Department of Health Policy and Management, Rollins School of Public Health, Emory University, Atlanta, GA USA; 5 Department of Pediatric Advanced Practice Nursing, Nell Hodgson Woodruff School of Nursing, Emory University, Atlanta, GA USA; 6 Department of Behavioral, Social, and Health Education Sciences, Rollins School of Public Health, Emory University, Atlanta, GA USA; 7 Division of Pediatric Surgery, Department of Surgery, Northwestern University Feinberg School of Medicine, Ann & Robert H. Lurie Children’s Hospital of Chicago, Chicago, IL USA; 8 Division of Gastroenterology, Department of Pediatrics, Northwestern University Feinberg School of Medicine, Ann & Robert H. Lurie Children’s Hospital of Chicago, Chicago, IL USA; 9 Division of Gastroenterology and Hepatology, Department of Medicine, Northwestern University Feinberg School of Medicine, Chicago, IL USA; 10 Department of Neurology, Biological Sciences Division and Center for Healthcare Delivery Science and Innovation, University of Chicago, Chicago, USA

## Abstract

**Background:**

Enhanced recovery protocols (ERPs) are an evidence-based intervention to optimize post-surgical recovery. Several studies have demonstrated that the use of an ERP for gastrointestinal surgery results in decreased length of stay, shortened time to a regular diet, and fewer administered opioids, while also trending toward lower complication and 30-day readmission rates. Yet, implementation of ERPs in pediatric surgery is lagging compared to adult surgery. The study’s purpose was to conduct a theory-guided evaluation of barriers and facilitators to ERP implementation at US hospitals with a pediatric surgery service.

**Methods:**

We conducted semi-structured interviews at 18 hospitals with 48 participants, including pediatric surgeons, anesthesiologists, gastroenterologists, nurses, and physician assistants. Interviews were conducted online, audio-recorded, and transcribed verbatim. To identify barriers and facilitators to ERP implementation, we conducted an analysis using deductive logics based on the five Active Implementation Frameworks (AIFs).

**Results:**

Effective practices (usable innovations) were challenged by a lack of compliance to ERP elements, and facilitators were having standardized protocols in place and organization support for implementation. Effective implementation (stages of implementation and implementation drivers) had widespread barriers to implementation across the stages from exploration to full implementation. Barriers included needing dedicated teams for ERP implementation and buy-in from hospital leadership. These items, when present, were strong facilitators of effective implementation, in addition to on-site, checklists, protected time to oversee ERP implementation, and order sets for ERP elements built into the electronic medical record. The enabling context (teams) focused on teams’ engagement in ERP implementation and how they collaborated to implement ERPs. Barriers included having surgical team members resistant to change or who were not bought into ERPs in pediatric practice. Facilitators included engaging a multi-disciplinary team and engaging patients and families early in the implementation process.

**Conclusions:**

Barriers to ERP implementation in pediatric surgery highlighted can be addressed through providing guidelines to ERP implementation, team-based support for change management, and protocols for developing an ERP implementation team. Future steps are to apply and evaluate these strategies in a stepped-wedge, cluster randomized trial to increase the implementation of ERPs at these 18 hospitals.

Contributions to the literature

- Despite evidence supporting enhanced recovery protocols (ERP), implementation of ERPs in children’s surgery is limited. Understanding the factors associated with ERP implementation across multiple surgery centers was essential to developing an implementation plan.
- Guided by the Active Implementation Frameworks, our research indicates there are modifiable barriers to ERP implementation in pediatric settings including the need for pediatric and family engagement, challenges based on surgical volume, and limited hospital-specific evidence of ERP effectiveness to drive stakeholders’ buy-in.
- These results provide insight into how to optimize ERP implementation for pediatric surgery.

## Background

Adult surgical disciplines have widely adopted enhanced recovery protocols (ERPs), evidence-based interventions that have been shown to reduce patient hospital length of stay, surgical complications, and costs and optimize patient post-surgical recovery [1–6]. Although there is some evidence of the use of ERPs in pediatric surgery, most reports are at a single institution or for a specific procedure [6–9]. Implementation of ERPs, more broadly, in pediatric surgery is lagging, even for specific ERP elements that have proven benefits. For example, despite the American Society of Anesthesiologists (ASA) recommendation for a clear liquid diet, followed by a 2-h preoperative fasting window, many hospitals still routinely counsel patients to have nothing to eat or drink after midnight of the day preceding a surgical procedure [10, 11].

**Fig. 1:**
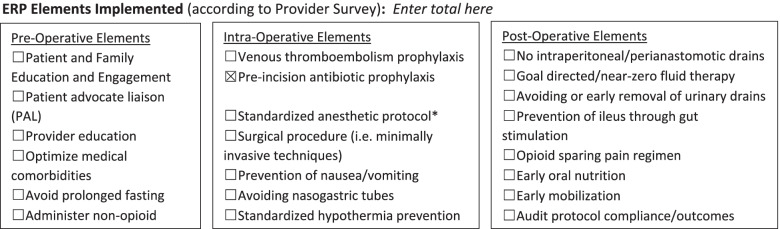
Enhanced recovery protocol (ERP) elements

A single-center study of an ERP for pediatric gastrointestinal (GI) surgery patients showed a steady increase in the number of ERP elements being used, over time, with a simultaneous decrease in length of stay; in median time to resumption of a regular diet; and in median doses of intraoperative and postoperative opioids and a trend toward lower complication and 30-day readmission rates [6]. Yet, in a recent study to evaluate the adoption of 21 ERP elements (Fig. 1) in GI pediatric surgery at 18 pediatric hospitals across the United States (US), on average, only 6 of 21 elements were widely adopted [12]. Implementation of a comprehensive ERP can reasonably be expected to be challenging, as ERPs consist of a large number of elements, spanning the pre-, intra-, and post-operative process, requiring practice changes at the hospital, department, and practice levels, and by a multitude of clinicians. Previous studies about ERP implementation have evaluated implementation barriers, processes, and outcomes in adult surgical settings [13–15]. To date, however, no comprehensive study about ERP implementation has been conducted in pediatric surgery. Such evaluations are important because of the distinct characteristics of the pediatric surgical environment compared to the adult environment. The long-term goal is to achieve similar outcomes and benefits from the implementation of ERPs in a pediatric population. To achieve this goal, it is necessary to understand the nuances of implementation in a new setting and, in this case, a pediatric setting [9]. The purpose of the current work is to present the perspectives and experiences of pediatric surgical teams. The perspectives of patients and families are presented separately [16]. The complexity of comprehensive ERP implementation and the lag in the uptake of its elements highlight the importance of using a systematic implementation framework to change practice comprehensively and proactively.

We conducted a formative qualitative assessment of barriers and facilitators to ERP implementation in 18 pediatric hospitals with a pediatric surgery service for pediatric patients undergoing GI surgery. Expected operations in a pediatric population ileocecectomy, partial/total colectomy, proctectomy/j-pouch, and ileostomy reversal, as well as laparoscopic techniques; these vary in complexity. We chose to use the National Implementation Research Five Network’s Active Implementation Frameworks (5 AIFs) [17], to identify which strategies can be designed to optimize comprehensive ERP implementation in pediatric surgery. The rationale for selecting the 5 AIFs is based on the Active Implementation Formula [18], which states that to achieve equitable outcomes, three key factors, effective practices, effective implementation, and enabling contexts are needed [17]. Equitable outcomes refer to focused attention on the culture, history, values, and needs of the community during the implementation process [19].

The 5 AIFs have been used to facilitate the implementation of programs to improve child well-being in various settings [20, 21] but have not yet been rigorously applied in pediatric surgery. By identifying barriers and facilitators within each of the 5 Active Implementation Frameworks (5 AIF), we aim to inform and optimize the planned implementation of ERPs in a multi-center trial to implement an ERP for pediatric GI surgery.

## Methods

### Sample

This study includes a purposive sample of pediatric surgery teams with a surgeon championing ERPs. We conducted online, web-based semi-structured interviews with clinicians and staff involved, or likely to be involved, in the use of an ERP at 18 diverse hospitals with a pediatric surgery service across the US. The hospitals were selected based on their planned participation in a future multi-center implementation trial to improve comprehensive ERP implementation [22]. The interviews were conducted as part of a mixed-methods baseline assessment of ERP use at the hospitals. Hospitals were in urban and rural settings and included pediatric surgery services within freestanding children’s hospitals and nested within adult hospitals. The study protocol was evaluated by the Institutional Review Boards at Ann & Robert H. Lurie Children’s Hospital of Chicago, Children’s Healthcare of Atlanta, Emory University, and Kaiser Permanente Georgia and considered exempt from full review.

### Data collection

**Fig. 2:**
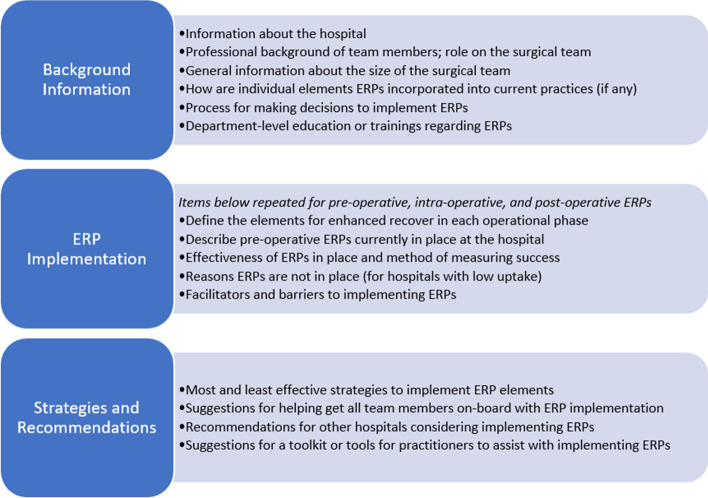
Interview guide structure for surgical team interviews

A semi-structured interview guide (Fig. 2) was developed, based on the results of a previously administered survey at the 18 hospitals about their use of pre-, intra-, and post-operative ERP elements [12]. The interview guide included questions about hospital and clinician characteristics, processes for GI surgeries, perceived barriers and facilitators to ERP implementation, and successful strategies and recommendations for future ERP implementation. ERP adherence and measurement practices from the prior survey [12] were compiled into a hospital-specific summary and used to initiate the interview, provide a definition of ERPs, and discuss the use of the individual ERP elements in the pre-, intra-, and post-operative stages. The guide also addressed barriers and facilitators to implementing each element and sought recommendations for further enhancement of ERPs. The interview guide was pilot tested and refined with one pediatric surgeon with experience in ERP implementation.

We conducted semi-structured, group interviews between October and December 2019 with clinicians and staff from each of the 18 hospitals participating in the collaborative study. Each interview lasted approximately 60 min. Interviews were facilitated by experienced interviewers who used best practices to elicit diverse viewpoints from participating surgery team members [23]. Interviews were conducted with groups with at least two interviewees participating per hospital. All participants were consented verbally; interviews were audio-recorded and transcribed, verbatim.

### Theoretical framework

**Fig. 3:**
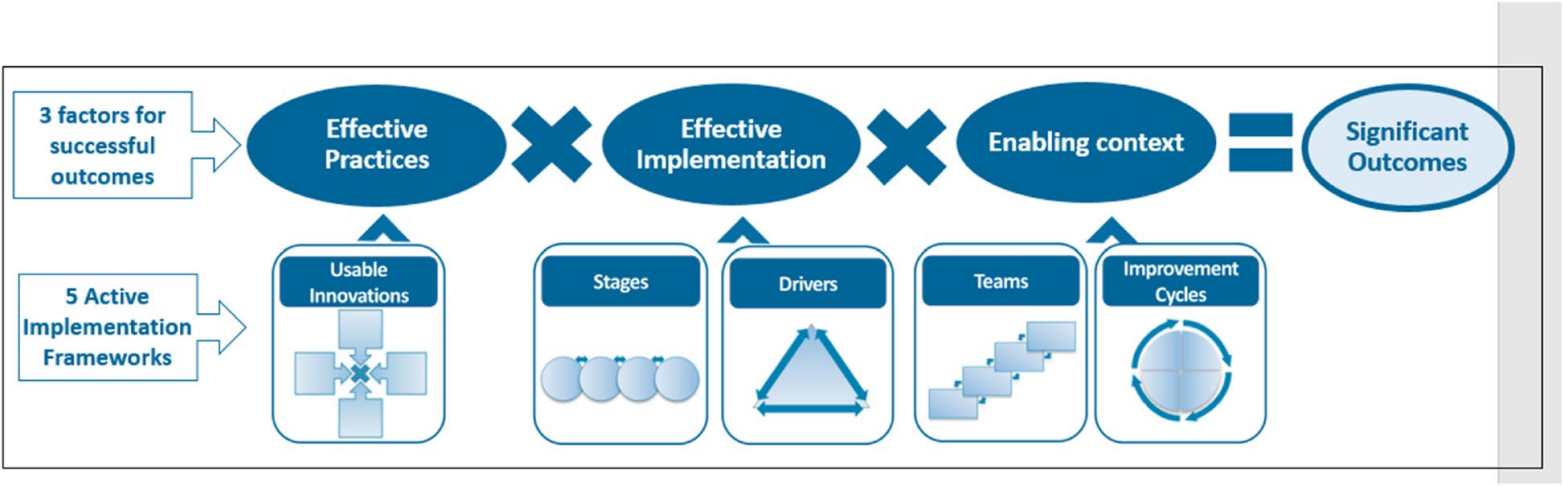
Active implementation formula [16]

The National Implementation Research Network identified 5 AIFs focusing on best practices for implementation and includes suggested mechanisms and strategies for the three key factors to achieve the desired outcomes [17, 24–26]. In the Active Implementation Formula [18] (Fig. 3), successful implementation is predicated upon having effective practices, effective implementation, and enabling context to achieve desired outcomes. To achieve *effective practices*, the *Usable Innovations* framework outlines criteria that emphasize having well-operationalized innovations that are teachable, learnable, doable, and readily assessable in practice. To achieve *effective implementation*, the *Stages* framework emphasizes the non-linear process starting with exploration and ending with full implementation of an innovation into practice. Furthermore, the implementation *Drivers* framework includes factors that support and enable successful implementation (e.g., developing competencies). Finally, the *enabling context* is determined by the role of *Implementation Teams* and *Improvement Cycles* [17]. We used these 5 frameworks to guide our analyses. Table 1 describes the 5 AIFs and provides definitions of how each framework was operationalized and applied to ERP implementation.

**Table 1:** Five Active Implementation Frameworks (5 AIFs) as applied to enhanced recovery protocol (ERP) implementation

	Framework	Description	Definition of framework components as applied to ERP	Strategies to address barriers and facilitators
*Effective practices*	Usable innovations	Well-operationalized innovations that are teachable, learnable, doable, and readily assessed in practice	Operationalization: essential functions of ERPs and pathways Fidelity: statements on fidelity to ERPs; may include measurement of implementation or recommendations and suggestions about measurement Function: evidence in other areas that have used ERPs or strategies for using ERPs; adaptation of ERPs in current practice Philosophy: attitudes and beliefs about ERPs; rationale for implementing ERP	• Evidence-based ERPs with validation by expert panels • Implementation tools
*Effective implementation*	Stages	Integrated, non-linear process starting with exploration and ending with full implementation of an innovation into practice	Exploration: descriptions of whether ERP implementation was feasible; readiness for implementation; activities related to preparing to implement ERPs, including engaging colleagues and experts Installation: discussions of steps needed prior to being able to implement ERPs at site, including capacity building; partnering with experts to build competencies to implement ERPs at site (i.e., seminars, trainings); consulting expert partners and consultants to implement ERPs at the site Initial implementation: experiences that hospitals have with the initial implementation of the innovation; early improvements or changes needed to the early ERP implementation; initial or preliminary results/outcomes or policies related to initial ERP implementation Full implementation: discussions about ERP implementation becoming standard practice at the site including standardization of protocols	• Local team infrastructure and defined roles: surgical champion, anesthesia champion, child life specialist, patient advocate liaison, quality improvement leader (QI), and ERP coordinator • Learning collaboratives for pediatric surgical hospitals
	Implementation drivers	Drivers of success including development of competencies, obtaining organizational supports, and engaging leadership.	Organization drivers: infrastructure components necessary to ERP implementation, including decision support data systems, and facilitative administration Competency drivers for clinicians: coaching and professional development designed to help the team use ERPs as intended; training that is skills-based and informed by adult learning processes; onboarding staff specifically to support ERP implementation Leadership drivers: support by hospital leaders for ERPs	• Monthly training curriculum through learning collaboratives • Coaching by topic experts • Facilitative leadership • Support engaging team members and hospital administrators
*Enabling context*	Teams	Supportive teams to define infrastructures and support methods and improve outcomes	Receptiveness and buy-in: receptiveness of ERPs among team members Collaboration: communication across and within departments, meetings about ERPs Team engagement: team members that have or should be involved in the implementation of ERPs, including a champion, health care clinician, data collector, patient and family liaison, and hospital administration liaison	• Toolkit and troubleshooting support to help hospitals move through the stages; with exploration completed, the learning collaborative will help hospitals move from installation and initial implementation phases to full implementation • ERP implementation sustainability assessment
	Improvement cycles	Based on Plan, Do, Study, Act (PDSA) process with rapid cycle feedback for continuous QI and learning	Not applicable in this preliminary examination of ERP implementation in the study centers; all centers were in the pre-implementation phase that precedes improvement cycles	• Quarterly data-driven feedback sessions for hospitals during learning collaborative meetings • QI expert on implementation teams

### Data coding and analyses

The transcripts were fully de-identified by removing all participant and hospital names, with only the professional role (e.g., nurse, surgeon) of participants being retained in the transcripts.

To identify barriers and facilitators of ERP implementation, we conducted a hybrid form of textual analysis using deductive logics [27, 28] by applying predetermined qualitative codes, based on the 5 AIFs’ mechanisms and strategies. A team of four experienced qualitative analysts (TD, WS, SB, SC) coded relevant data as either a “barrier” or “facilitator” to the implementation of an ERP if the interviewee referred to the item as such. They developed the codebook by operationalizing each AIF by seeking examples from one randomly selected transcript. The coding team repeated this process until no new codes emerged, which occurred after coding transcripts from 4 hospitals. Table 1 summarizes the codebook definitions of the components within each framework, as applied to ERPs in pediatric surgery. The remaining interviews were concurrently coded in dyads to identify the AIF codes and the barriers and facilitators. The data were then organized to examine the barriers and facilitators for each AIF. Two research team members (TD and WS) discussed each framework and the associated barriers and facilitators to confirm inductive coding consistency and reconciled any differences [29]. MaxQDA 2020 (VERBI Software, 2019) [30] was used to support data storage, coding, and analysis.

## Results

We conducted 18 semi-structured interviews with 48 participants including surgeons (*n* = 24), anesthesiologists (*n* = 10), a gastroenterologist (*n* = 1), nurse practitioners (*n* = 5), research specialists (*n* = 6), a physician assistant (*n* = 1), and a clinical pharmacist (*n* = 1). Table 2 provides quotations of each identified barrier and facilitator, categorized by the 5 AIFs. Some additional exemplary quotes are provided in-text. The research team identified the barriers and facilitators for each of the 5 frameworks, except for the “Improvement Cycle” framework, which was expected, as the hospitals were all in the pre-implementation phase that precedes improvement cycles since no pediatric surgery services were comprehensively implementing the full enhanced recovery protocol, defined as all 21 elements. We characterized the barriers and facilitators to the implementation of pediatric ERPs into three key factors: effective practices, effective implementation (implementation stages and implementation drivers), and enabling contexts based on the Active Implementation Formula [17].

**Table 2:** Description of barriers and facilitators of enhanced recovery protocol (ERP) implementation along the 5 Active Implementation Frameworks (5 AIFs) and components

Framework	Component	Barrier	Facilitator
**Usable innovations (effective practices)**	Operationalization	Surgeon: “And I think also **training the surgeons**. You’re going to have to wait to have a second IV. You’re going to have to wait for that block to happen. Adding that to our turnover time or our anesthesia preparedness time so that we can appropriately block that operative slot.”	Surgeon: “I’ll add that the success I’ve seen us do with the Pectus surgery was really **getting down to the specifics and standardizing things**. [ … ] So, for our group I think if we leave any kind of leeway, then there’s a chance that it won’t be followed. But **if we give specifics** that this is exactly how we do it **for each one**, then that’s **more successful**.”
	Fidelity	Anesthesiologist: “And they re-looked at the data to how many people were **being compliant with all the elements** of the pathway and it dropped **down to like 40%**, and so having to go back and even though everyone knows what they’re supposed to do, you get a little bit lax and what’s kind of happening, and so when everyone’s busy clinically it’s hard to go back and having to remind people.”	Not identified as a facilitator
	Function	Not identified as a barrier	Surgeon: “And then secondly, I think any kind of resource we put into it, whether it’s a toolkit or so forth, is how easily that will be galled and utilized for other diagnoses. **Because if we’re using for a small subset of 50 patients a year, it’s hard to maintain**.”
	Philosophy	Not identified as a barrier	Surgeon: “ ..first making sure that [ … ] you have everybody .. being a part of the decision-making, and [ … ] **making sure that patients and families are an important component of the whole process** and that we don’t underestimate. [ … ] So I think that the family and the patient is an under-utilized resource[ … ] a lot of the **programs** that we try to implement **would be far more effective**.”
**Stages (effective implementation)**	Exploration	Surgeon: “I think that **there’s no specific barriers**. It’s just a **matter of actually implementing** those things and **making it a priority**.”	Surgeon: “We’ve had some **training from adult colorectal surgeon who is working on ERP** from their side of things, and they came to our group, one of our group meetings, and had some **training on the ERP occurring** in the adult side of things, and **gave us some great ideas** that we can do from the pediatric side.”
	Installation	Surgeon: “And one of the challenges that we identified early on is **identifying all the right stakeholders** and **getting buy-in** broadly amongst the providers along the entire continuum of care.”	Surgeon: “I guess first making sure that [ … ] you have **everybody at the table** so that you’re not forcing someone to do something without being a **part of the decision-making**.”
	Initial implementation	Surgeon: “And then you have these **hiccups in the workflow**. So, I think that **tracking those measures** is kind of the next step. I’ve been spending the last year, year and a half, with the help of kind of multiple team members trying to put this into place, but it **hasn’t been** a totally like, **here’s a checklist** guys, let’s go.”	Interviewer: “Probing for what intraoperative elements are easier to implement” Surgeon: “The **ones that I have direct control over**. Whether they get prophylaxis, antibiotics, surgical technique, avoiding NG tubes, those are all **easy to implement, because I’m the one kind of driving the ship.”**
	Full implementation	Surgeon: “if it **can be applicable to thousands of our patients**, then I think that would be **easy to put more resources towards**.”	Surgeon: “For our adults on the post-operative side, we have **basically checklists and guidelines printed** out in our workroom, and so all the residents have that. And then we also have little **ERAS order sets in EPIC** that we can order for a post-op patient as well. So it’s quite simple.”
**Implementation drivers (effective implementation)**	Organization drivers	Surgeon: “The part that it gets **a little bit harder** is the, the **transition from the outpatient discussion to the inpatient side of things** [ … ] the inpatient order set is super-duper easy to do. The **outpatient order set is a little bit harder** to do when we **rely** really more **on our multidisciplinary care team**, our pharm D to kind of make that happen. [ … ] I wish there was like one order set that was easy to order in clinic that did everything.”	Surgeon: “We’ve put together basically, a **power point slide, which kind of lists the workflow**, as far as this is what they get for preoperative, this is what they get day prior, intraoperative, postoperative, discharge plan. And that is available for our team, saved on a drive that they have access to. [ … ] it’s **in multiple places for reference**, and then it’s kind of **built into our order sets**.”
	Competency drivers for clinicians	Surgeon: “I think one of the barriers is that **we don’t have a specific team of nurses**. We have lots of nurses and the hospital’s **philosophy overall is that nurses should be trained in everything**. [ … ] we have people who preferentially want to be in one room or another, but that doesn’t always work.”	Surgeon: “We have to try to work with them and **convince them and show the evidence**. Or, compromise and kind of decide, “Okay, well, we’re not going to do what I want. We’re not going to do what you want. We’ll do somewhere in the middle, so that both of us...” But, in general I think that there’s **been a lot of good buy-in**.”
	Leadership drivers	Surgeon: “There’s a little **bit of unfortunate politics and drama involved in leadership** roles within the people that would make sense in my mind to play a role in this. So that needs to get sorted out and we’re working on it.”	Surgeon: “I think it **takes resources**. It takes people being **given time away from clinical duty** for either a very long day of planning or multiple repeated meetings. I think it takes administrative support.”
**Teams (enabling context)**	Receptiveness and buy-in	Surgeon: “We have the 30 anesthesiologists on faculty, and every day I could be with any one of them [ … ] Some of them are a little bit like – **the data isn’t really clear about this, why are we doing it**. And so there’s just been, depending on who I’m with, I may get a lot of engagement, or I may get... I think it’s just one of those things that’s **going to take time to evolve. Physicians are not always easy to change**.”	Surgeon: “Yeah, I would kind of agree that by and large there **is fairly high institutional buy-in** towards any of these **quality improvement** things, especially ones that don’t cost money [ … ] Sometimes there’s an issue if you want to implement a pathway, and surgeons kind of have their own ways of doing things. We **have to try to work with them and convince them and show the evidence**. Or, compromise [ … ] But, in general I think that there’s been a lot of good buy-in.”
	Collaboration	Surgeon: “It **highlighted to us the areas where we don’t have consensus amongst the surgeons** and as far as components of the pathway, as well as consensus amongst the anesthesiologists for components of the pathway.”	Surgeon: “For us to choose an ERA approach, it’s a **multidisciplinary input usually triggered by a discussion between the physician and the anesthesia pain team**, but that case is usually identified by the surgeon saying, “I need to operate on this IBD patient.””
	Team engagement	Anesthesiology: “Yeah. So we have a large surgical operation here and I think just what MD_10_1 is saying**, we have to get so many different people on board**. There’s **literally 95 different anesthesiologists** that **might be in the room on a given day**.”	Surgeon: “I guess first making sure that, as you even mentioned **you have everybody at the table** so that you’re not forcing someone to do something without being a part of the decision-making, and [ … ] I think that the number one thing is **making sure that patients and families are an important component** of the whole process and that we don’t underestimate. [ … ] I think that the family and the patient is an under-utilized resource. If there is a way to figure out how to tap into that resource, I think that a lot of the programs that we try to implement would be far more effective.”

### Effective practices: usable innovations framework

The usable innovations framework emphasizes making practices effective by making them teachable, learnable, doable, and readily assessed in practice [17]. Participants reported a lack of training on ERP elements among team members as a barrier to the operationalization of ERPs. Reported facilitators to ERP implementation included having clear definitions of ERP elements and early inclusion of patients and families in the implementation process.

Measuring fidelity of ERP implementation was identified as a consistent barrier for hospitals. Indeed, no hospital reported the existence of a system to track ERP implementation or associated clinical and patient outcome measures. One surgeon discussed only having an informal process for monitoring outcomes:

... I haven’t been really good at making sure preoperatively [that] they’ve been getting or following their preoperative orders. But [ … ] I try to do kind of like a social round after their surgery, and kind of see how things are going, making sure they’re getting the instructions from our wound team, and then our dieticians have this ostomy eating plan that they can take home, and kind of refer to as needed. [Surgeon]

The absence of any monitoring system was attributed to a lack of resources and institutional support. Participants at hospitals with low surgical volume discussed their “insufficient power” to conduct outcome analyses with limited data. One participant noted that ensuring the team understood their role in ERP implementation was an operational barrier, stating:

It’s just operationalizing it in a way everybody understands their role and how to do this. [Anesthesiologist]

None of the reported barriers could be characterized as part of the function framework component. Rather, functional resources were identified as facilitators, including the ability to leverage existing processes from other departments, such as adult surgery, or from hospitals with higher volumes that use ERPs.

I think it’s identifying those things so that you’re not recreating the wheel, and it’s resources that the nurses are used to using. [Surgeon]

Participants reported a key philosophy to facilitating ERP implementation is the belief ERPs lead to improved patient outcomes. Participants emphasized that using available evidence to inform the end-user perception of ERPs’ clinical benefit and establishing buy-in was essential to facilitating ERP implementation. Participants further discussed the importance of establishing goals for ERP implementation, including alignment with an institution’s desire to improve quality of care, such as decreased length of stay, minimizing opioid use, avoiding postoperative ileus, decrease patient nausea, or more promptly resuming a regular diet and maintenance of patient homeostasis.

### Effective implementation: implementation stages framework

The implementation stages framework takes teams through the different stages of effective implementation, including exploration, installation, initial implementation, and full implementation [17]. Study participants identified specific barriers to exploration, including a lack of prioritization of ERPs at the hospital, resistance among clinical partners (e.g., surgeons, anesthesiologists) due to a lack of familiarity with ERPs, and physicians needing time to adjust to ERPs in practice. Reported facilitators included having experts available within the hospital in non-surgical specialties (e.g., gastroenterology, pain specialists) and national collaborators available to provide training to individual, hospital-based ERP implementation teams.

Reported installation barriers included challenges with identifying and involving all stakeholders in the ERP onboarding process. Starting the ERP implementation process was reported as comparable to “moving the battleship” given the number of moving parts involved in getting a pediatric surgical service ready to implement an ERP. Reported facilitators included having an ERP champion with protected time to prepare the team for ERP implementation.

Reported barriers of the initial implementation stage included a lack of workflow or implementation tracking, variation in ERP implementation, difficulties with identifying barriers to implementation, low [surgical] case volumes leading to an inability to study the effectiveness of the ERP at the hospital, and slow adaptation in medications used for pediatric surgery patients. One surgeon noted:

there’s variability in the utilization of nasogastric tubes, there’s variability in the utilization of early post-operative feeding. There’s variability in the opioid usage post-op. And I’d say there’s probably even variability in the choice of pre-operative antibiotic prophylaxis. [Surgeon]

Reported facilitators of the initial ERP implementation included early stakeholder engagement from staff, as well as family and patient engagement during protocol development. Participants also shared that it is easier to control the implementation of ERP elements that are within their clinical purview or job description. Implementation was also reported to be facilitated by using the hospital’s own data to develop the protocol,

protocols that are based on our own outcomes have been very easy, [Surgeon].

Participants reported specific barriers to full implementation, including a lack of standardization or protocols in place, variation in patient needs, low case volume limiting the hospitals’ ability to standardize protocols, and hospitals over-reliance on traditional practices:

if it isn’t broke, let’s not fix it. And sort of holding on to the traditional, I’ve done this the same way for 10 years and there’s no reason why I should change it now, [Anesthesiologist].

Participants reported that when surgeons have complex patients with challenging operative cases, they are more likely to abandon ERPs and revert to their traditional practices. Reported facilitators of full implementation included having a protocol available for distribution in multiple formats and discussing the protocol in a multidisciplinary team setting.

### Effective implementation: implementation drivers framework

The implementation drivers framework includes the development of competencies, obtaining organizational supports, and engaging leadership to lead to effective implementation [17]. Participants reported that organizational drivers (e.g., electronic health record (EHR) systems to flag or remind providers of ERP elements) were not available at some hospitals and represented a major barrier to creating order sets in the EHR or to identify ERP cases, electronically. Participants also noted barriers to ERP implementation during transitions between phases of care, from pre- to intra- to post-operative settings. For example, order sets for ERPs may not be transferrable between inpatient and outpatient settings (Table 2)*.* Participants indicated the availability of decision support systems could facilitate ERP implementation through using an alert in the EHR to identify patients eligible for ERPs, making ERP checklists available, electronically, for eligible patient encounters, generating a standardized ERP clinical decision support tool in the EHR, and using the EHR for process and outcome measure tracking.

The major barrier reported for competency drivers was staff turnover. For example, trainees (e.g., residents, fellows) and nurses rotating onto the service and not specifically part of the GI surgical team may require ongoing training and orientation to become familiar with the ERP. One surgeon noted:

I think one of the barriers is that we don’t have a specific team of nurses. We have lots of nurses and the hospital’s philosophy overall is that nurses should be trained in everything. So yes, in general we have people who preferentially want to be in one room or another, but that doesn’t always work. And so that would be infrequent ... Nurse infrequent with the process, has to have discussion about that. Although, they’re trying to put the right people in the room. [Surgeon].

Participants also noted the need to train and coach surgeons and anesthesiologists to conduct specific ERP procedures (e.g., regional blocks, perioperative analgesic adjuncts), while reported facilitators of ERP implementation included educating all providers on ERPs, having reciprocal education between different groups of clinicians (e.g., from nurses to surgeons and from surgeons to nurses), and using evidence as part of clinician education being key.

Participants reported barriers to leadership drivers, including a lack of leadership buy-in to support ERP implementation. They reported the need to navigate “office politics” to ensure leadership support and resource investment including personnel necessary to measure outcomes. Reported facilitators to implementation included administrative support, protected time for staff and clinicians to implement ERPs, and institutional buy-in to the ERP culture, all of which are more likely to occur when there are little-to-no costs related to implementation. Similarly, several participants brought up hospital and department size as a potential barrier to ERP implementation. Specifically, they reported that for hospitals and departments with low surgical patient volume, it is difficult to implement education/training due to the infrequency of surgical cases. Participants indicated that pediatric surgery services that are a “wing” in an adult hospital have difficulty implementing innovations specific to pediatric surgery.

One barrier which is that we are a children’s hospital within an adult hospital … So, we have to think in terms of our patients the early standpoint, because we are sharing preop, postop with the adult population, so we have to go an extra mile to make sure things which we want for pediatric patients are implemented we follow up on those things. [Anesthesiologist]

### Enabling context: teams framework

The teams framework emphasizes having teams define infrastructures and support strategies and improve outcomes; this creates a foundation or enabling factors for ERP implementation [17]. Receptiveness and buy-in were identified as key barriers and facilitators to ERP implementation. Key reported barriers included a lack of institutional support, paucity of hospital-specific outcome data, difficulty in identifying the right stakeholders at their hospitals, variation in levels of baseline knowledge and consensus about of ERPs, and lack of clinician receptiveness for specific ERP elements (e.g., early enteral nutrition). Reported facilitators related to receptiveness and buy-in included having outcomes data demonstrating how ERPs could reduce hospital costs and resource utilization, standardized procedures and protocols for clinicians to follow, and ERPs in other surgical procedures as a reference and leveraging leadership support to garner buy-in from more clinicians.

Collaboration among team members was noted by participants to be hindered if there was a lack of team consensus on ERPs and poor communication between clinicians. Reported communication barriers could preclude collaboration both within a profession (e.g., among surgeons) and across professions (e.g., surgeons, nurses, anesthesiologists). However, having strong relationships within and across teams and clinician groups was reported to foster collaboration and facilitate implementation. Participants highlighted examples of having regular meetings to discuss ERP implementation broadly, and ERP cases, specifically. Clearly defining and communicating the workflow within and across teams was another aspect reported as facilitating collaboration. Some participants from smaller pediatric surgery teams reported being able to access each other quickly by telephone with questions and other teams mentioned having recurring meetings to facilitate communication. As one surgeon highlighted, team engagement is a step in the right direction but not a panacea:

So it really highlighted to us that even when you have engaged family and you know the institutions are moving towards this pathway, that there are hurdles. [Surgeon]

Team engagement barriers included lack of communication among teams, inadequate team composition, and team size. Team engagement was reportedly hindered by the lack of an established ERP protocol. Some participants reported inadequate team composition and lack of engagement of critical team members, such as an ERP representative, patient liaison, child life specialists, and outcomes data collectors. Other participants reported that a large team size would be more challenging to engage in the ERP implementation process.

Participants endorsed that engaged teams would be a facilitator to ERP implementation. Early engagement of patients and families, presence of services that were supportive of personnel on the team, and having a coordinator to monitor ERP cases throughout the care pathway were important facilitators. Furthermore, the existence of ERP champions (e.g., executive sponsor, surgical lead, anesthesia lead) and a multidisciplinary team (e.g., surgery, nursing, pain management) would facilitate ERP implementation.

## Discussion

In-depth interviews with 48 pediatric clinicians and staff caring for pediatric surgical patients at 18 hospitals across the US provided insight into the potential barriers and facilitators to the implementation of comprehensive ERPs in pediatric surgery. Guided by the 5 AIFs [17, 20, 21, 31], our analysis identified several barriers and facilitators are unique to pediatric surgery practice implementation, including the need for pediatric patient and family-specific engagement, challenges of limited pediatric surgical volume, barriers related to pediatric services embedded in an adult hospital with different priorities, and limited available evidence of ERP effectiveness to drive buy-in (e.g., medications). Our study also provided needed insights on the implementation process which was a gap in the existing literature [32].

In the Active Implementation Formula [18], successful implementation is predicated upon having effective practices, effective implementation, and enabling context to achieve desired outcomes. Applying this framework in our analysis suggests that pediatric surgical services will need to implement evidence-based ERP protocols that are data-driven (effective practices), and clearly identify roles for team members. Further, success will be contingent upon providing consistent training and support to engage and prepare teams (effective implementation) and providing toolkits to enable implementation and data-driven feedback during the learning process (improvement cycles). Patient and family engagement, data collection and tracking, training and education, surgical volume and practice size, buy-in from stakeholders, and having an ERP champion were dominant implementation drivers for successful ERP implementation in pediatric surgery. This study extends our understanding of barriers and facilitators to ERP implementation for a diverse and complex population not previously studied, namely pediatric surgical patients [33]. Prior studies in adults identified barriers of team and administrative buy-in, team receptiveness [32], team engagement, resources, inter-department collaboration, and standardization of order sets.

Patient and family representation and buy-in were identified by study participants as important components throughout the process of implementing ERPs in pediatric GI surgery. Opportunities to involve patients and families include involvement in developing ERPs and introducing patients and families to ERPs prior to surgery. Participants noted the need for administrative support to actively engage families and patients as implementation team members. Including patient and family members on the implementation team can be used as an implementation strategy to co-produce strategies to solve issues around adherence (e.g., drinking a carbohydrate liquid prior to surgery) [34]. Patients and families were identified as an important part of the care pathway for the co-production of educational materials and tools supporting implementation. Furthermore, patients and families need to be introduced to ERPs in an early phase of care (e.g., preparation prior to surgery for different steps and phases of engagement throughout the perioperative pathway). Such engagement has been modeled at pediatric hospitals successfully implementing ERPs and includes preoperative counseling to introduce patients and families to the “why” of ERPs, the daily goals involved in ERPs, and expectations for a successful discharge and return to baseline activity after surgery [35].

Although data collection, specifically surrounding ERP adoption, was noted as important to successful ERP implementation, all participants reported that their hospital had substantial barriers to data tracking. No hospital was reported to have a formal tracking system in place that could be used for monitoring ERP implementation, noting the lack of human resources and clinical tools to properly track which ERP elements were implemented and the frequency of implementation. Several key ERP elements cannot be easily abstracted through a retrospective review of an EHR, highlighting the need for prospective data collection. Formal audits of ERP implementation are the best way to monitor fidelity [36, 37] and can also be used to increase protocol adherence [38]. Hospitals would benefit from having systematic processes developed to measure adherence to ERP elements based on established evidence.

Training and education were also identified as essential components to ERP implementation and sustained compliance [39, 40]. In addition to achieving standardization, training is important to engage all team members throughout the perioperative continuum. Training surrounding ERPs was reported as necessary to garner buy-in from all hospital stakeholders including hospital administration and clinicians. Training on and use of data-driven outcomes were endorsed as methods to reduce resistance to ERPs among colleagues and hospital leadership. Engaging leaders across departments, including surgery, nursing, and anesthesia should precede resource allocation for ERP implementation. Using data to enhance support has also been identified as an important facilitator in other studies of ERP implementation among adult surgical populations [13, 14]. Hospitals expressed support for use of their own outcomes data to enhance receptivity. Furthermore, extending training to all team members is a barrier when all teams do not have a specific team of ERP clinicians, but rather a rotation of team members. This is a well-known challenge encountered within modular care delivery systems, such as nursing [41, 42]. Potential strategies to overcome this barrier include the assignment of a “nurse coordinator” and efforts to centralize patients on one surgical floor. The nurse coordinator can help disseminate critical information to peers, guide the patient throughout the process, reinforce the importance of various ERP elements, and clarify misunderstandings [38]. Similarly, dissemination to peers can be facilitated by appointing designated surgeons and anesthesia champions.

Finally, low case volumes of pediatric GI surgeries were a major reported concern. In some hospitals, low case volume limits their ability to use internal data for education and to generate institutional buy-in. Hospitals with low surgical volume would benefit from having access to clinical outcomes data specific to pediatric surgery from other hospitals (individual or compiled from multiple hospitals). Because the percentage of patients undergoing non-urgent gastrointestinal surgery procedures is generally low in each hospital, clinicians (e.g., surgeons and anesthesiologists) may not identify a patient as a candidate for many of the aspects of the ERP. Furthermore, the low case volume makes it difficult to ensure standardization of the process and conduct of rapid improvement cycles. Lastly, sustaining the implementation of a complex ERP can be a challenge if only applied to a small number of patients. One potential solution is to look for opportunities to horizontally integrate ERPs to other patient populations who can benefit from ERPs. This allows the surgical team to increase the volume of surgeries performed using ERPs and hospitals to achieve economies of scale.

As highlighted by a surgeon participant, ERP implementation is a team effort that requires numerous factors to be set into place before the ERP elements can be implemented, measured, and assessed. Successful implementation needs to go beyond checking off the boxes of the protocol. The qualitative interview data presented in this manuscript, in combination with survey data from the 18 hospitals [12], are being used to inform an evidence-based toolkit and the development of protocols for ERP implementation. Individual hospitals do not have the resources, time, or administrative support to both develop protocols and standardize procedures. Stakeholder-informed practices strengthen healthcare implementation strategies [43], and the results of this qualitative work will inform the development of a learning collaborative for surgical teams and tools for engaging patients and families. This study expands our understanding of the need for implementation strategies to include perspectives and insights from clinicians, patients, and families. These shared, in-depth understandings contribute to modifications that are meaningful and useful in the implementation process for surgery and other clinical applications as demonstrated by Deatrick et al. whose work utilized stakeholder influence to guide modifications for the implementation of psychosocial screening in pediatric cancer [43]. Future research is needed to explore more multiple stakeholder-informed implementation strategies; these processes can be applicable across additional clinical areas of practice and other patient populations.

A strength of this research is the rich, descriptive data that provides a better understanding of ERP implementation in pediatric surgery and includes participants who may be key stakeholders in the future implementation of ERPs in varied hospital settings. Having interviews with surgical teams, compared to one-on-one interviews, provided a more expansive perspective on barriers and facilitators to ERP implementation. Each surgical service had an ERP champion to help recruit other team members; in this purposive sample, the champions helped identify other team members with working knowledge to discuss their team’s implementation. Group interviews implemented using best practices can generate discourse [44], but one limitation is it may inhibit team members from sharing their personal viewpoints. This sampling plan also means this work may not be applicable to pediatric surgical services in precontemplation or without plans to implement ERPs. Those surgical services may have different barriers to implementation that are not represented in this research.

Another strength is the applicability of this process to other surgical practices and patient populations. Following the process of engaging key stakeholders and identifying needs and opportunities informs the future incorporation of stakeholder input into implementation strategies, while our work focused on implementing an intervention accepted in adult surgery yet underutilized in pediatric surgery. Recognizing similar disparities in practices can be addressed by engaging key stakeholders to understand gaps and next steps. Active stakeholder engagement is more important than ever when we think about implementing interventions in pediatric groups—not just in surgery—because this is truly a complex patient population with unique needs. Small sample sizes [43] and low surgical volume are concerns hindering our ability to study ERP implementation in pediatrics. This manuscript presented part of a body of work that served as the formative intervention development process. In addition to this surgical team input, we previously conducted a baseline assessment of ERP implementation [12]. A separate manuscript from this work also highlights the patient and family experience of ERPs [16]. This ecological framework for understanding implementation recognizes the interconnectedness of the patient, family, providers, and health system perspectives. The patient’s needs (individual) must be addressed through a multi-level system of intrapersonal support (family), community (PALs), systems (hospital/providers; data systems), and policy (hospital leadership setting policy to support ERP implementation).

The next steps in this research are to conduct a multi-site, randomized, controlled trial, ENhancing Recovery In CHildren Undergoing Surgery (ENRICH-US), that supports protocol development and implementation of an ERP for pediatric GI surgery patients and evaluates both the implementation of the ERP and its effectiveness on patient outcomes [22, 33] Standard, primary outcomes for ERP implementation aligns with previous research and include the 21 ERP elements, hospital length of stay, and readmission; we will also assess patients’ return to activity, post-surgery nutrition plan, and pain level experiences. From the identified strategies in Table 1, we will incorporate the following strategies to increase ERP implementation in pediatric surgical settings: (1) development of implementation strategies and a toolkit targeting these barriers and enhancing facilitators of implementation, (2) use of a learning collaborative approach to implement an ERP across 18 pediatric surgery services, and (3) implementation a uniform ERP measurement system and measuring fidelity of implementation.

## Conclusion

This study comprehensively and systematically applied the 5 AIFs to identify barriers and facilitators of ERP implementation. Ultimately, using this theoretical foundation to code and analyze the data has facilitated the identification of potential strategies for achieving successful ERP implementation in pediatric settings. Based on the Active Implementation Formula (Fig. 3), we have identified strategies to support effective practices (evidence-based ERPs and implementation tools), effective implementation (local team infrastructure support including team member roles and responsibilities; a learning collaborative for pediatric surgery groups with monthly support, coaching and training by topic experts, tools to engage key stakeholders), and enabling contexts (a toolkit to support implementation, sustainability assessments, quarterly data-driven feedback sessions, and access to a QI expert on the implementation teams). This study establishes a theory-driven, contextual understanding of barriers and facilitators to ERP implementation in hospitals serving pediatric patients undergoing GI surgery. The current study was able to inform and optimize the next steps, which are to apply and evaluate these strategies in a future stepped-wedge, cluster randomized trial to increase implementation of ERPs in 18 pediatric surgery centers, which will allow us to determine if the strategies identified in this study will be able to increase ERP implementation.

## Data Availability

The data that support the findings of this study are stored with Northwestern University, but restrictions apply to the availability of these data, which were used under license for the current study, and so are not publicly available.
